# Evaluation of the Therapeutic Efficacy of Sequential Therapy Involving Percutaneous Microwave Ablation in Combination with ^131^I-Hypericin Using the VX2 Rabbit Breast Solid Tumor Model

**DOI:** 10.1371/journal.pone.0120303

**Published:** 2015-03-23

**Authors:** Miao Zhu, Xiao-An Lin, Xiao-Ming Zha, Wen-Bin Zhou, Tian-Song Xia, Shui Wang

**Affiliations:** Departments of Breast Surgery, The First Affiliated Hospital of Nanjing Medical University, Nanjing, Jiangsu, China; Taipei Medical University, TAIWAN

## Abstract

**Purpose:**

Combination of percutaneous microwave ablation (PMWA) and intravenous injection of ^131^I-hypericin(IIIH) may bear potential as a mini-invasive treatment for tumor. The objective of this study was to assess the effect of PMWA and IIIH in breast tumor growth.

**Methods:**

Ten New Zealand White rabbits bearing VX2 breast carcinomas were randomly divided into two groups (each 5 examples) and processed using PMWA followed by IIIH and IIIH alone. The IIIH activity was evaluated using planar scintigraphy, autoradiography and biodistribution analysis. The maximum effective safe dose of IIIH was found through 48 rabbits with VX2 breast tumor, which were randomized into six groups (n=8 per group). Subsequently, a further 75 rabbits bearing VX2 breast solid tumors were randomly divided into five groups (each 15 examples) and treated as follows: A, no treatment group; B, PMWA alone; C, IIIH alone; D, PMWA+IIIH×1 (at 8 h post-PMWA); and E, PMWA+IIIH×2 (at 8 h and at 8 days post-PMWA). The therapeutic effect was assessed by measurement of tumor size and performation of positron emission tomography/computed tomograph (PET/CT) scans, liver and renal function tests and Kaplan-Meier survival analysis.

**Results:**

The planar scintigraphy findings suggested a significant uptake of ^131^I in necrotic tumor tissue. The autoradiography gray scales indicated higher selective uptake of IIIH by necrotic tissue, with significant differences between the groups with and those without necrotic tumor tissue (*P*<0.05). The maximum effective safe dose of IIIH was 1mCi/kg. The PET/CT scans and tumor size measurement suggested improvements in treatment groups at all time points (*P*<0.01). Significant differences were detected among Groups A, B, D and E (*P*<0.05). Lower levels of lung metastasis were detected in Groups D and E (*P*<0.05). There were no abnormalities in liver and renal functions tests or other reported side effects.

**Conclusion:**

IIIH exhibited selective uptake by necrotic tumor tissue. Sequential therapy involving PMWA+IIIH was successfully inhibiting tumor growth and prolonging survival.

## Introduction

Overall survival was not significantly different between breast conserving treatment and radical mastectomy in stage II breast, which was verified by EORTC 10801 trial [1]. Breast conserving surgery is currently accepted as the therapy of choice by numerous surgeons and patients. It has been confirmed that breast conserving surgery followed by postoperative breast adjuvant radiotherapy is the normal treatment for proper patients with early breast tumor. However, the cosmetic results and complications that occur after the use of the standard breast-conserving therapy protocol, which consists of lumpectomy and a 6-week adjuvant radiotherapy course [2–4], pose a major concern[5]. Therefore, there is a need for a more minimally invasive treatment protocol.

Compared with traditional surgical therapy, the image-guided ablative therapies, which contain microwave ablation, radiofrequency ablation, laser ablation, high-intensity focused ultrasound and cryoablation, have distinct advantages that cover superior cosmetic results, less complications and mortality, cooperativity with other cancer treatments, the capacityto complete ablative treatment in an outpatient basis, convenience for simultaneous imaging monitoring, smaller expense of treatment and reproducible[6]. Compared to the other thermal ablative modalities, percutaneous microwave ablation (PMWA) has particular advantages, such as preferable heating of cystic masses, bigger tumor ablation range, uniformly higher temperatures inside the tumor, shorter ablation duration, advanced convection profile, capicity to use multiple applicators and minor pain[7–10]. At present, PMWA has been extensively used for the treatment of tumors located in the lung, liver, bone, adrenal gland and kidney. Moreover, duo to the special anatomical location of breast where is between ectopectoralis and skin, microwave ablation is more suitable for breast tumor than others solid tumors. We have proved that US-guided PMWA is a promising treatment for solitary breast cancers [11].

Similar to the standard process that breast conservation therapy has to be followed by intraoperative or postoperative radiotherapy[4], the microwave ablation also has to be followed by a postoperative radiotherapy or others ionization therapy. Compared to conventional postoperative adjuvant radiotherapy, intraoperative radiotherapy has the probability to be a hopeful modality for early breast cancer treated using breast conserving therapy, attributable to decrease of the normal tissues exposure to radiation, reduction of the radiotherapy duration and results in a lower local recurrence rate[12,13]. Tumor necrosis therapy (TNT) which was first reported by Epstein et al. was that ^131^I-labeled monoclonal antibodies to intracellular antigens, which are integral structural components and are retained by degenerating cells, can kill the viable tumor cell around the necrosis tumor tissue[14]. Therefore, the use of tumor necrosis therapy (TNT) after the completion of the microwave ablation may has the same effect as intraoperative radiotherapy.

Hypericin that is the main component of *hypericum perforatum* has been widely used in folk medicine. Hypericin can be extracted from the common St. JohnsWort (*Hypericum species*), as well as be synthetised from the anthraquinone derivative emodin. Hypericin was recently shown to be an agent exhibition a specific affinity for necrotic tissue that is potentially useful for tumor necrosis therapy (TNT) [15]; Therefore, radiolabeled hypericin can be used with targeted radiotherapy to target the surviving tumor tissue adjacent to necrotic tissue [15], exerting an effect similar to that of intraoperative radiotherapy following surgical resection.

Collectively, therapeutic efficacy of sequential therapy involving percutaneous microwave ablation in combination with ^131^I-hypericin was evaluated by a rabbit VX2 breast solid tumor model.

## Materials and Methods

### Experimental design

**Fig 1:**
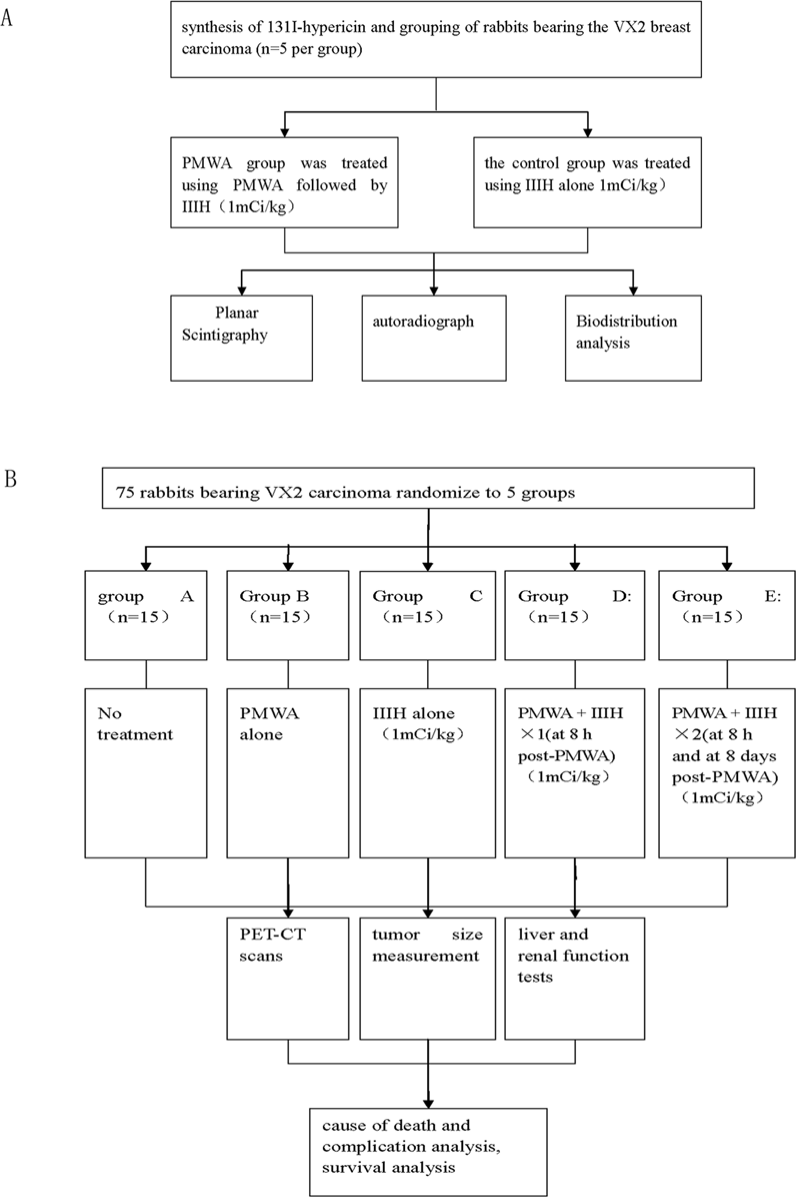
Flow chat of this study. (A) The verification of ^131^I-hypericin affinity to necrotic tumor tissue. (B) Evaluation of the therapeutic efficacy of sequential therapy involving percutaneous microwave ablation (PMWA) in combination with intraveneous injection of ^131^I-hypericin (IIIH) using the VX2 rabbit breast solid tumor model.

Initially, 10 rabbits bearing the VX2 breast solid tumor were randomly divided into two groups (each 5 examples) by picking numbers out of a hat; PMWA group was treated using PMWA followed by injection of ^131^I-hypericin (IIIH), and the control group was treated using IIIH alone. Subsequently, planar scintigraphy, biodistribution analysis and autoradiograph were performed to verify the affinity of IIIH for necrotic tumor tissue (Fig. 1A).

To find the maximum effective safe dose of IIIH, 48 rabbits bearing VX2 solid tumors were randomized into six groups (n = 8 per group). Then the rabbits pretreated by PMWA were treated by IIIH of various density (0.125mCi/kg, 0.25mCi/kg, 0.5mCi/kg/kg, 1mCi/kg, 2mCi/kg and isometric PBS) at 8 hours after PMWA. Positron emission tomography/computed tomography (PET/CT) was fulfilled to three rabbits, which were randomly choiced from every group by picking numbers out of a hat, to assess the theraputic effect at days 4, 8 16 after IIIH. Additionally, white blood cell count of all rabbit from every group was detected to assess the reaction in each group at 24 hours, 48 hours, 72 hours and seven days after IIIH.

To evaluate the effects of sequential therapy involving PMWA and IIIH on tumor growth, a further 75 animals were randomly divided into five groups (each 15 examples) by picking numbers out of a hat as follows: A, no treatment group; B, the PMWA alone group, with an ablation zone that covered the tumor and was monitored using two-dimensional ultrasound; C, the IIIH alone group; D, PMWA followed by a single IIIH treatment via the ear vein at the dose of 1mCi/kgat 8h after PWMA; and E, PWMA followed by two IIIH treatments via the ear vein at a dose of 1mCi/kg at 8h and 8 days after completion of PMWA. PET/CT was subsequently fulfilled to assess the therapeutic effect in each group prior to therapy, and at days 4, 8 and 16 after therapy. The Kaplan-Meier method was used for survival analysis. (Fig. 1B)

In the study, n refers to the number of animals, with one acquisition from each rabbit, with mean and standard deviation obtained from each group used for each protocol (five or fifteen animals each group in respective experiment part).

According to the literature [16,17], experimental animals grouping and sample size were determinated.

### Animal model

All experimental procedures were approved by the Institutional Animal Care and Use committee of Nanjing Medical University and conformed with Guide for the Care and Use of Laboratory Animals: Eighth Edition. In our study, Adult female New Zealand White rabbits weighing 2.0–3.0 kg were employed. The VX2 cell line used in this study was gained from the Surgery Department of our hospital. At first, the VX2 tumors that was cryo conserved and gained from the Surgery Department of our hospital were thawed. Second, the thawed VX2 tumors were cut into pieces and innoculated to the upper leg musculature of a New Zealand White rabbit to produce the tumor carrier rabbit from which the tumor material was taken after the innoculated tumor was formed. Third, the tumor tissue was cut into small strips (1.5 × 1.5 × 6 mm) and was then injected in subcutaneously underneath the right second nipple using a 16-gauge needle. All of the above process were performed after the rabbit was anesthetized through an intravenous injection of pentobarbital sodium (0.8 to 1.2 ml/kg at a concentration of 30 mg/ml; Sigma, St. Louis, MO, USA) [18]. The rabbits were treated when the tumor size reached 2.0–2.5cm in diameter, as monitored using calipers at 30 days post-inoculation. If tumors had not reached or had exceeded the required size range by this time, they were excluded from further study prior to treatment. The rabbits were monitored every 2–3 days and were allowed to live to the humane endpoint when they had lost >10% of their initial body weight. Rabbits were sacrificed using intravenous injection of pentobarbital sodium (3.5 to 4ml/kg at a concentration of 30 mg/ml; Sigma, St. Louis, MO, USA) when the endpoint came.

Every rabbit was raised in a standard cage with 45 cm height and 0.28 m^2^ floor size in a temperature of 22°C and humidity of 55±5%. And the day and night alternation per 12 hours was provided with lights on at 8:30pm. And 15 fresh air changes per hour was performed.

### Drug preparation

^131^I-hypericin was produced using a standard iodogen method with a labeling yield of >92% [19], which was determined by ascending paper chromatography. The specific activity of ^131^I-hypericin was 900MBq/mg; For the sake of intravenous injection at various dose, the ^131^I-hypericin was diluted in water and poluethylene glycol 400 (volume ratio, 80:20);

### PMWA protocol

The microwave system (Nanjing Yigao Microwave Institute, Nanjing, China) consisted of a microwave generator and a hollow water-cooled-shaft antenna, a flexible coaxial cable and a microwave generator. For this study, an output power of 40W and an electromagnetic field of 2.5MHz were selected. The duration of microwave irradiation was 2.5 min, over which almost all of the tumor was ablated. After the same anesthesia as prepration of animal model was completed, the rabbits were positioned supine and sterilized. Subsequently, the water-cooled-shaft antenna (2mm in diameter) was penetrated into the tumor along its long axis, guided by two-dimensional ultrasonography.

### *In Vivo* Planar Scintigraphy

After radioiodination with a labeling efficiency of approximately 92%, the uptake of IIIH by necrotic tumor tissue was visualized *in vivo* by means of single-photon emission computed tomography (SPECT) scanning (SKYLight ECT, Philips, The Netherlands). Images were gained at 2 h post-injection of IIIH in two standard projections (anterior and posterior) after the rabbit was fixed on the camera bed in the supine position. A window of 20% centered on the energy peak of ^131^I (364 keV) and a matrix of 128 ×128 pixels were selected. Imaging data were obtained for 15 min.

### Autoradiography

In the case of each tumor collected at 24 h post injection, all specimens were snap-frozen with optimum-cutting-temperature compound (Sakura Finetek, Torrance, CA, USA) and sectioned into 6–8 consecutive 5-mm slices. Subsequently, the sections were stained for autoradiography and dried at 40°C in open air. Subsequently, the sections were exposed and photographed on BAS-SR 2025 Fuji phosphorous film (Fuji Medical Systems, Hanover Park, IL, USA), which was then scanned through the FLA5100 Multifunctional Imaging System (Fuji Medical Systems, Hanover Park, IL, USA).

### Biodistribution

The rabbits were scrificed using intravenous injection of pentobarbital sodium (3.5 to 4ml/kg at a concentration of 30 mg/ml; Sigma, St. Louis, MO, USA) immediately after the SPECT imaging session. Lung, liver, kidney, heart, spleen, brain, stomach, intestine, blood, bone, muscle and tumor tissues were removed and weighed; radioactivity was measured using a Cobra γ-counter (Packard Cobra, MN, USA). The uptake of IIIH in the various organs was assessed as the T/NT [T and NT represent the uptake of IIIH by the target organ and the non-target organ (e.g., muscle), respectively].

### Measurement of tumor size

The rabbit weight and tumor sizes (measured by means of calipers) were recorded prior to treatment and on days 4, 8 and 16 after treatment. Tumor volume was then calculated in mm^3^ according to the approximation V = 1/2*ab* ^2^, where *a* is the long axis and *b* is the short axis of the tumor in mm [20]. The tumor size change ratio was assessed by means of the value of the tumor size post-treatment divided by the tumor size prior to treatment in respective groups.

### *In Vivo* PET/CT experiment

After anesthetized, the rabbits were immobilized in the supine position with their axis coincidence with the PET scanner in the center of the field (Biograph 64 TruePoint PET-CT: Siemens, Munich, Germany). At 1 h after an ear vein injection of fluorodeoxyglucose ^18^F-FDG at a dose of 14.8 MBq/kg, the rabbits received a prone scan for 20 min. The specific parameters were as follows: a slice thickness of 5 mm; 120 kV; 80 mA; and 7 min per bed position. Before 18F-FDG PET/CT scanning, all the selected tumor-bearing rabbits must be fasting for at least 6 h. The standardized uptake values (SUV) were determined.

### Measurement of hepatorenalfunction and white blood cell count

Auto-biochemical analyzer (Roche, Basel, Switzerland) was performed to measure the hepatorenal including glutamic-pyruvic transaminase (GPT), glutamic-oxaloacetic transaminase (GOT), blood urea nitrogen (BUN) and creatinine. White blood cell count was performed by blood cells analyzer. (Beckman Coulter, California, USA) Blood samples were collected from the marginal ear vein before and after treatment (i.e. on days 8, 16 and 32).

### Statistical analysis

SPSS 13.0 software (SPSS, Inc., Chicago, IL, USA) was applied to statistical analysis. Quantitative data were expressed as the mean ± standard deviation. The independent samples t-test was applied to statistical analysis of the autoradiography gray scale and biodistribution between the PMWA+IIIH and the IIIH alone groups.

Analysis of variance (ANOVA) was performed for comparison of repeated measurement data among the various groups, namely GPT,GOT, BUN, creatinine, SUV, white blood cell count and the tumor change ratio. If the Mauchly test of sphericity was found to be significant, we used the Green house-Geisser correction. In cases where significant differences were revealed by means of ANOVA, a least significant difference (LSD) was performed for pairwise comparison.

The Kaplan-Meier method was used for survival analysis. *P* values <0.05 were considered as existence of statistics difference.

## Results

The animals’ health status was monitored throughout the experiments by a health surveillance programme according to Institutional Animal Care and Use Committee (IACUC) guidelines [21]. The rabbits were free of all viral, bacterial, and parasitic pathogens listed in the IACUC recommendations.

### The confirmation of the maximum effective safe dose of IIIH

**Fig 2:**
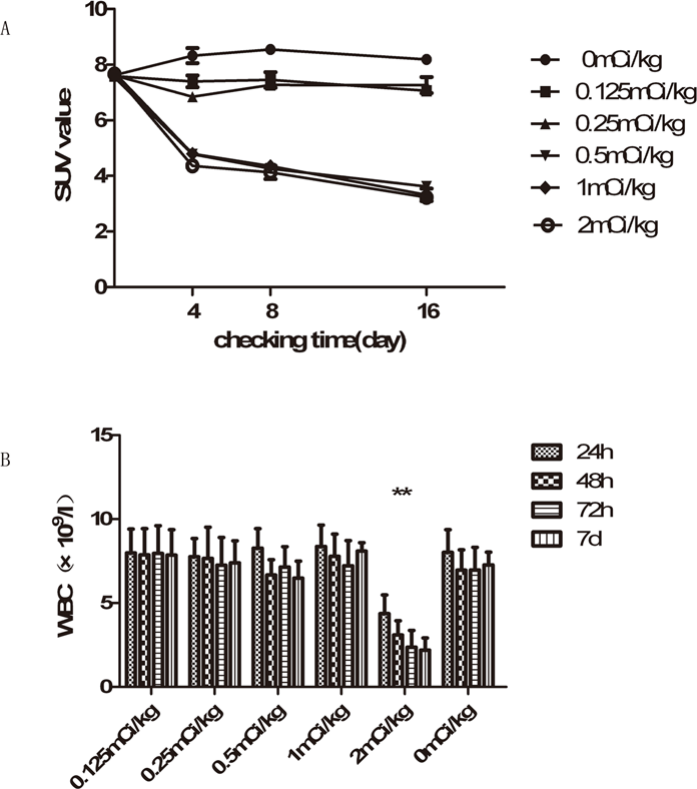
Changes of SUV (standardized uptake value) and white blood cell count in each treatment group with various density IIIH. (A) Compared with the other groups, the larger dose groups (0.5mCi/kg, 1mCi/kg, 2mCi/kg). (B)The white blood cell count in 2mCi/kg group is apparently less than any of the other groups (** represents the presence of statistic, *P*<0.01).

As shown in Fig. 2A, the treatment groups all have the apparent therapeutic effect (F = 472.117, *P*<0.01), which was revealed by repeated ANOVA measurements of the SUV for all the indicated time points. The three groups of which dosage are more than 0.5mCi/kg have the better therapeutic effect than the other groups, which were demonstrated through LSD test. And there is no statistics differences among the three groups of which dosage are more than 0.5mCi/kg (*P*>0.05).

As shown in Fig. 2B, the apparent decrease of white blood cell count appeared when dosage of IIIH arrived 2mCi/kg, which was demonstrated through LSD test. Therefore 1mCi/kg of IIIH was confirmed as the maximun effective safe dose.

### Assessment of IIIH uptake using Planar Scintigraphy and Autoradiography

**Fig 3:**
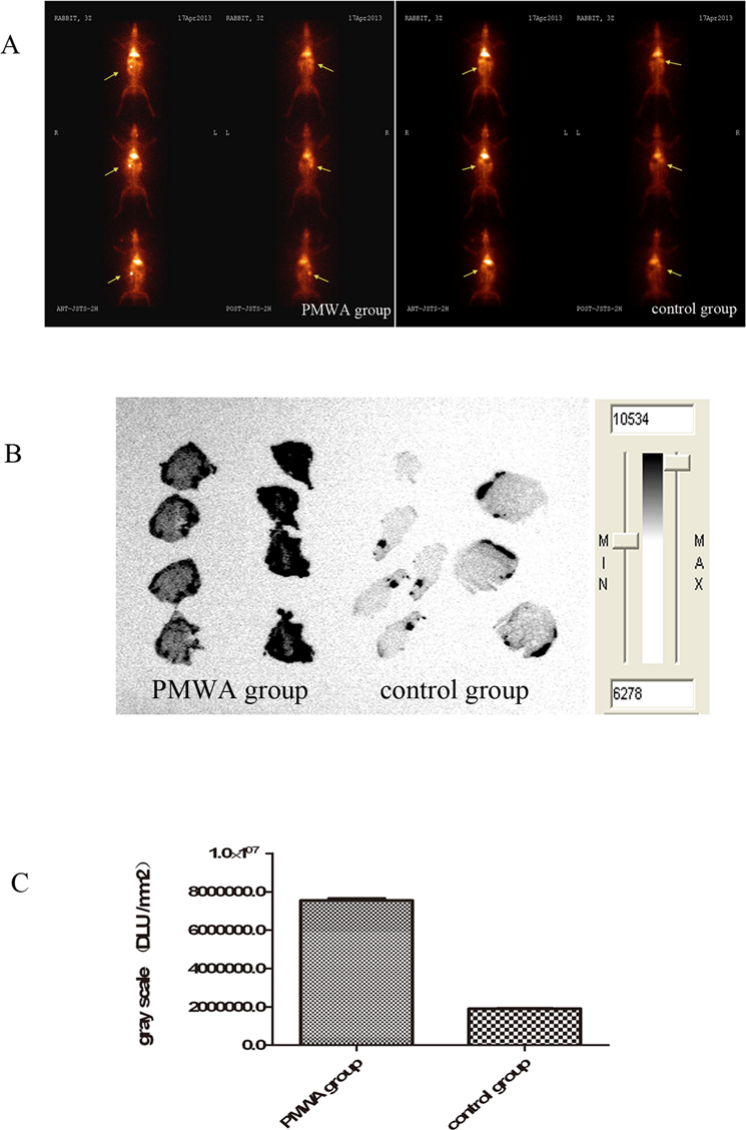
Differences in uptake of ^131^I-hypericin in the control group and PMWA group. (A) In vivo planar scintigtaphy. The arrows indicate the VX2 tumors, which had been inoculated underneath the right second nipple. The radioactivity overtly accumulated in the necrotic tumors in the PMWA group; By contrast, no radioactivity accumulated in the tumors in the control group. (B and C) The gray scale regarding IIIH was significantly higher. There was a significant difference in the gray scale between the control group and the PMWA groups.

Planar scintigraphy was performed to achieve optimal imaging; the uptake of IIIH was detected at 2 h after injection. The group treated with PMWA exhibited high uptake of IIIH at necrotic tumor sites; however, the group that was not treated using PMWA exhibited no uptake of IIIH at the tumor sites. Representative images are shown in Fig. 3A. Autoradiography was used to obtain evidence of IIIH selectivity for tumor necrosis (Fig. 3B). The gray scale was significantly higher (7.55×10^6^±8.59×10^4^) in the PMWA treatmentgroup compared to that in the groupthat did not undergo PMWA treatment (1.91×10^6^±1.12×10^4^) (t = 117.278; *P*<0.05; Fig. 3C).

### Biodistribution of IIIH

**Fig 4:**
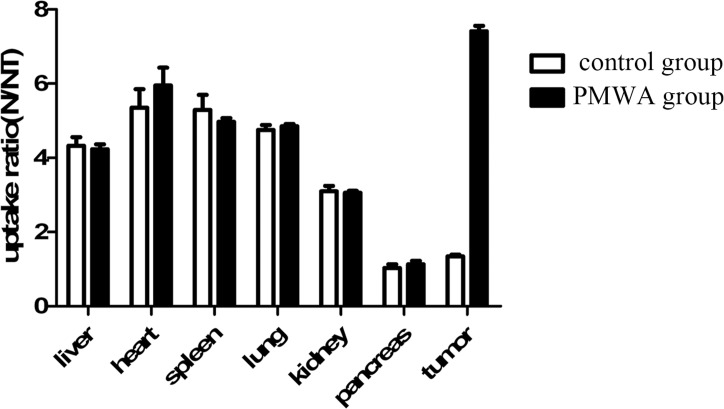
The biodistribution of ^131^I-hypericin in PMWA group and control group. The uptake of ^131^I-hypericin by the tumor was significantly higher in the PMWA group (7.41±0.15) compared to that in the control group treated with IIIH alone (1.34±0.05) (t = 40.989; *P*<0.05). No significant difference was observed in normal organs uptake between the two groups. T, target; NT, non-target

At 135 min after injection of IIIH, the T/NT ratio was found to be significantly higher (7.41±0.15) in the groups that underwent PMWA treatment compared to that in the groups that did not (1.34±0.05) (t = 40.99; *P*<0.05). There were no differences in the T/NT for liver, spleen, heart, lung, kidney and pancreas between the groups that underwent PMWA treatment and those that did not (Fig. 4).

### Tumor size change ratio

**Fig 5:**
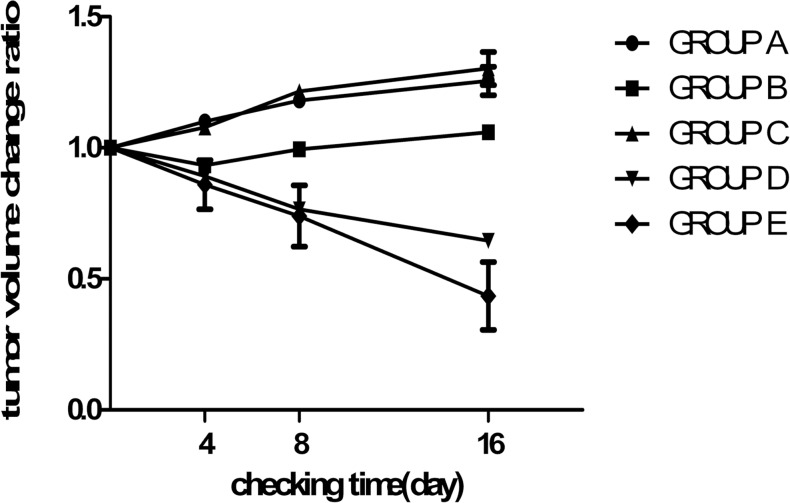
Change in tumor size with time after treatment. A statistically significant difference was observed between any two of the groups (*P*<0.05), with the exception of groups A and C. The tumor size decreased in Groups D and E, whereas it increased in groups A, C and B, after an initial decrease. The tumor shringage was more prominent in group E compared to that in group D.

Tumors had a pretreatment volume of 6373.7±1656.5 m^3^, with no significant difference in size among the groups. As shown in Fig. 5, there were apparent statistical differences for all treatment groups revealed by repeated ANOVA measurements regarding the tumor size change ratio (F = 124.48; *P*<0.01) at all indicated time points. Furthermore, there were significant time-group interactions regarding the tumor size change ratio (F = 52.70; *P*<0.01). The main effect of time exhibited statistically significant differences (F = 5.96; *P*<0.01).

Using the LSD test, significant differences were observed between any two groups (*P*<0.01), with the exception of GroupsA and C (*P* = 0.51 and *P*>0.05, respectively). In Groups A and C, the tumors exhibited an apparent trend to increase in size with time. Tumor size in Groups D and E decreased between days 0 and 16 after treatment using PMWA and IIIH, whereas tumor size in Group B gradually increased, after an initial decrease on day 4 post-treatment. Furthermore, the tumor size in Group E decreased more rapidly compared to that in Group D after day 8.

### PET/CT for the evaluation of the efficacy of sequential therapy

**Fig 6:**
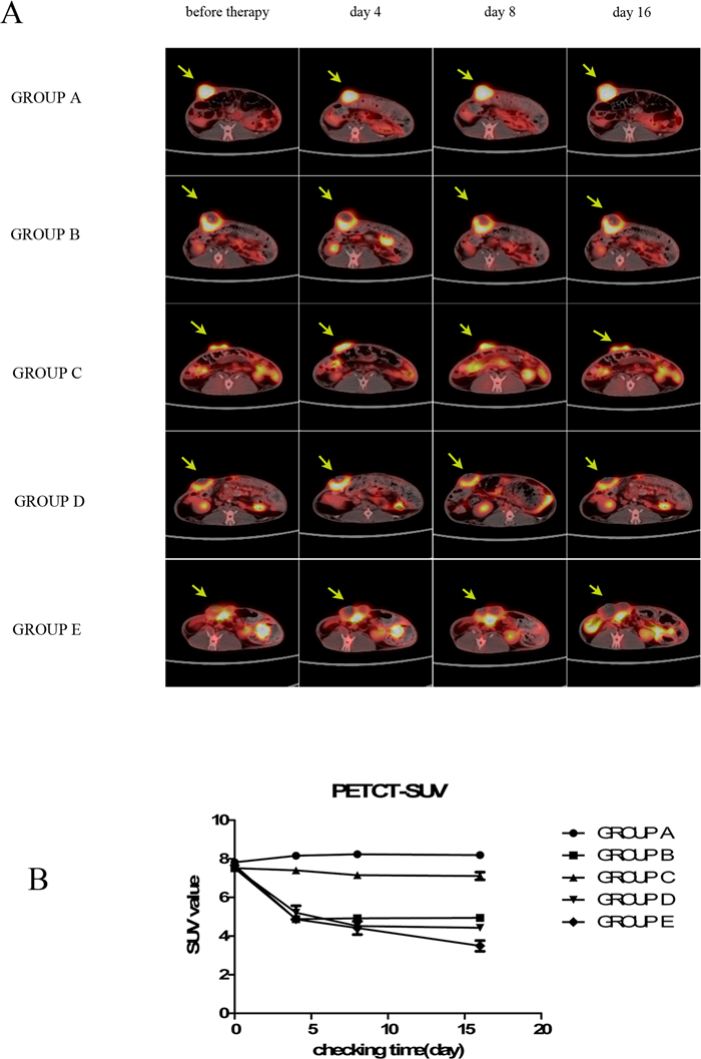
Representative results from positron emission tomography/computed tomography scans. (A) The arrows indicate the VX2 tumors, which were inoculated underneath the right second nipple. In groups B and D, flurodeoxyglucose (^18^F-FDG) uptake was clearly decreased and reached a plateau on day 4 and 8, respectively. In groups B, D and E, ring-shaped accumulation of ^18^F-FDG is observed at the site of percutaneous microwave ablaton treatment. (B) In Group E, ^18^F-FDG accumulation was decreased overtly between days 0 and 16 (*P*<0.01). SUV,standardized uptake value.

Representative images are shown in Fig. 6A. There were apparent therapeutic improvements in Groups B, D and E revealed by ANOVA regarding repeated measurements of the SUV for all the indicated time points (F = 314.59; *P*<0.01). As shown in Fig. 6B, there was a significant time-group interaction (F = 55.70; *P*<0.01). The main effect of time exhibited statistically differences (F = 286.48; *P*<0.01). A significant difference (*P*<0.01) was scaned through the LSD test.

There was no significant difference in SUV among groups prior to treatment. The SUV in Groups B, D and E had evidently decreased at day4 after treatment using PMWA. The SUV in Group B had no signigicant difference between days 4 and 16 after PMWA treatment. The SUV in Group D had no signigicant difference between days 8 and 16 after administration of PMWA. In Group E the SUV evidently decreased between days 0 and 16 after treatment using PMWA, whereas the SUV in Groups B and D gradually reached a plateau at days 4 and 16, respectively.

### Survival times of rabbits with VX2 breast solid tumor implants

**Fig 7:**
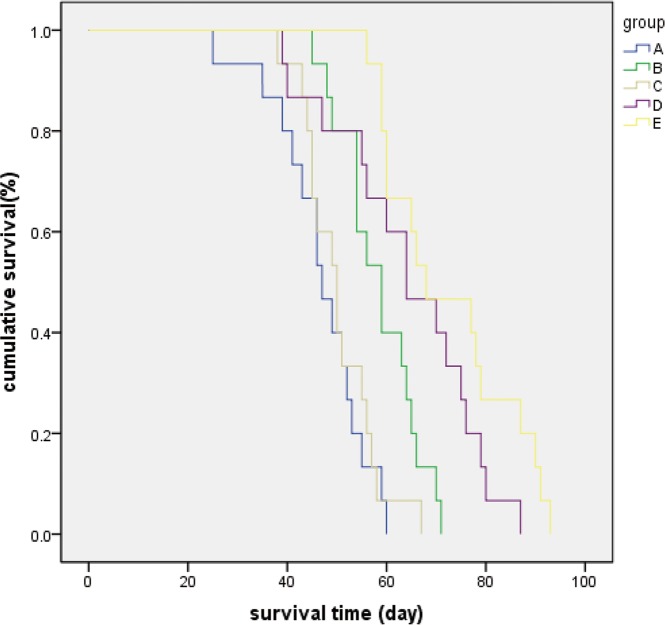
Kaplan-Meier survival curves and survival rates of rabbits with breast VX2 implantations in the five groups. Statistically significant differences were observed when comparing any two groups (*P*<0.01) with the exception of groups A and C (*P* = 0.543)

No significant difference was observed in the Kaplan-Meier survival curves for Groups A and C. The mean survival times of animals in Groups B, D and E were longer compared to that in Group A. The Kaplan-Meier curves revealed that survival times in Groups B, D and E where significantly higher than no treatment GroupA (*P*<0.05) (Fig. 7). The animals in Groups B, D and E survived for 12, 18 and 26 days, respectively, which was longer than no treatment group. There had no VX2 allograft-recipient rabbit survived for >60 days in Group A. The survival rate in Group E was 50% at day 66, which was the longest among the five groups (Fig. 7).

### Metastasis, complications and the results of the hepatorenal function test

The vast majority of tumor metastasis was mainly detected in the lung and lymph nodes, and occurred in 90% of the rabbits in Groups A and C, whereas the incidence of lung metastasis and lymph metastasis was significantly decreased in Groups D and E (table 1) (*P*<0.05). Most rabbits died of tumor lung metastasis.

**Table 1:** Tumor metastasis and complications after treatment in each group.

	Group A	Group B	Group C	Group D	Group E
**Lung metastasis**	14	11	13	8	4
**Lymph node metastasis**	13	12	14	7	5
**Tumor ulcerating**	12	7	12	6	4
**Puncture path infection**	0	0	0	1	0
**Gastrointestinal bleeding**	0	0	0	0	0

The tumor metastasis in lung and lymph node in group D and group E is less than in group A and group C (P<0.05). And tumor ulcerating in group D and group E is less than in group A and group C (P<0.05).

The tumor metastasis in lung and lymph node in group D and group E is less than in group A and group C (*P*<0.05). And tumor ulcerating in group D and group E is less than in group A and group C (*P*<0.05).

**Fig 8:**
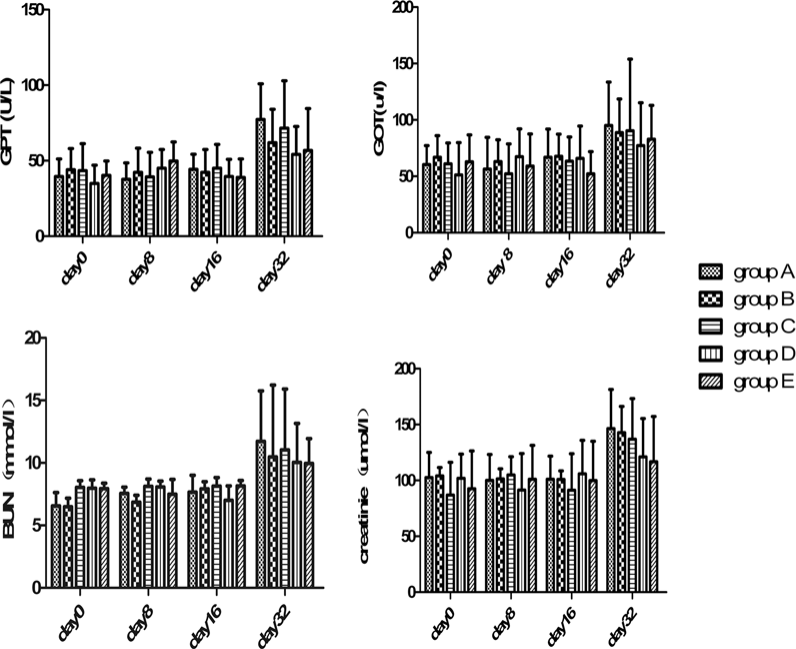
Changes in hepatorenal function. Changes inglutamic-pyruvic transaminase (GPT), glutamic-oxaloacetic transaminase (GOT), blood urea nitrogen (BUN) and creatinine levels with time after treatment; the levels had increased by day 32 after treatment in all groups. There was no significant difference among groups at any of the indicated time points.

None of the rabbits exhibited overt clinical signs of toxicity, for example, gastrointestinal bleeding, diarrhoea, and changes in hair or skin in response to treatment with PMWA or IIIH. No significant difference was observed regarding the liver and renal function tests among the five groups (*P*>0.05) (Fig. 8).

## Discussion

Currently, for ablative experiment, as most of the equipmentis not designed specifically for animal experiment but for human study, experimental animal size is a considerable problem. Thus, an animal model larger than the mouse or rat was required. The VX2 tumor is a highly malignant squamous cell solid tumor which has already been used widely for the study of local treatment for solid tumor, such as breast [18,22], lung [16], liver [23] and kidney [24]. The VX2 tumor is the only tumor model which is easy to be innoculated in larger animals like rabbit. Therefore, this tumor was selected as the model for use in our study. In addition, the result of this study has the general significance for others solid tumors.

In the present study, we demonstrated that treatment using PMWA followed by one or two administrations of IIIH at an effective dose resulted in a promising therapeutic effect in rabbits bearingVX2 breast tumors.

Our results are consistant with the previously reported research in a rodent bearing VX2 liver tumor, in which the residual tumor tissue was eliminated by the IIIH that targeted the necrosis tissue produced by vascular disrupting agent combretastatin A-4 phospate (CA4P)[23].

Concerning to TNT, relevant clinical trials that a ^131^I-radiolabeled monoclonal antibody was used to treat and diagnose solid tumors, such as lung and brain cancer, is currently under way in United States and China[14,25]. When compared to macromolecular agents, such as monoclonal antibodies, peptides or small organic molecules, small molecules such as hypericin exhibit superior permeability and a lower uptake rate by the reticuloendothelial system[23,26].

In the present study, hypericin was labeled with ^131^I to form ^131^I-hypericin, which emits both β and γ radiation. ^131^I-hypericinhas been used for the treatment of liver rhabdomyosarcomas[23]. ^131^I is a radioactive isotope with a relevant short half-life of 8 days and a 2mm β-particle penetration depth, which means higher safety and feasibility. Therefore, the use of IIIH followed by PMWA may exert an effect similar to that of intraoperative radiotherapy following dissection in breast cancer patients. And so on, each effective dose can cause a centrifugal inactivated range to residual viable tumor tissue.

It has been reported that FDG-PET was more sensitive than morphologic imaging in detecting breast residual tumor and recurrence [27]. In addition, ^18^F-FDG-PET is more applicable than the other evaluation methods on early assessment of treatmen in sensitivity and accuracy. In our study, the quick and significant decrease of SUV at VX2 tumor sites was observed soon after the effective treatment was implemented, which was shown in PET-CT scanning result.

The maximum effective safe dose of IIIH was found to be 1 mCi/kg body weight in our study. Routine tests for blood and hepatorenal function, measurement of weight and hematoxylin-eosin (HE) staining of liver tissue were performed to investigate the toxicity of IIIH (data not shown). When the IIIH dose was 1 mCi/kg, the body weight profile and routine tests for blood and hepatorenal function gave results that were in the normal range; abnormal changes in HE staining of liver tissue were not seen (data not shown).

There were certain limitations to our study. First, the mechanism underlying the affinity of hypericin for necrotic tumor tissue has not been fully elucidated. Second, the power at which PMWA is administered and the duration of treatment requires further optimization to achieve the optimal ablation area covering the entire tumor margin without normal tissue damage. Third, large animal breast tumor models are limited. Whether there is a similar therapeutic effect exists in other animal models remains to be determined.

In summary, in the rabbit VX2 breast solid tumor model, sequential therapy involving PMWA and IIIH decreased tumor growth and improved survival. As such, PMWA followed by IIIH bears great potential to replace traditional surgery, chemotherapy and radiotherapy as a primary curative therapy or palliative care therapy. Although further investigations are required to optimize the delivery parameters regarding PMWA and the dose of IIIH, this sequential therapy will likely continue to receive considerable attention as a minimally invasive treatment for malignancy.
